# The Effect of Renal Denervation on Plasma Adipokine Profile in Patients with Treatment Resistant Hypertension

**DOI:** 10.3389/fphys.2017.00369

**Published:** 2017-05-30

**Authors:** Nina Eikelis, Dagmara Hering, Petra Marusic, Jacqueline Duval, Louise J. Hammond, Antony S. Walton, Elisabeth A. Lambert, Murray D. Esler, Gavin W. Lambert, Markus P. Schlaich

**Affiliations:** ^1^Human Neurotransmitters and Neurovascular Hypertension and Kidney Disease Laboratories, Baker Heart and Diabetes InstituteMelbourne, VIC, Australia; ^2^Iverson Health Innovation Research Institute, Swinburne University of TechnologyMelbourne, VIC, Australia; ^3^School of Medicine and Pharmacology - Royal Perth Hospital Unit, University of Western AustraliaPerth, WA, Australia; ^4^Heart Centre Alfred HospitalMelbourne, VIC, Australia

**Keywords:** renal denervation, resistant hypertension, obesity, non-esterified fatty acids, adiponectin

## Abstract

**Background:** We previously demonstrated the effectiveness of renal denervation (RDN) to lower blood pressure (BP) at least partially via the reduction of sympathetic stimulation to the kidney. A number of adipocyte-derived factors are implicated in BP control in obesity.

**Aim:** The aim of this study was to examine whether RDN may have salutary effects on the adipokine profile in patients with resistant hypertension (RH).

**Methods:** Fifty seven patients with RH undergoing RDN program have been included in this study (65% males, age 60.8 ± 1.5 years, BMI 32.6 ± 0.7 kg/m^2^, mean ± SEM). Throughout the study, the patients were on an average of 4.5 ± 2.7 antihypertensive drugs. Automated seated office BP measurements and plasma concentrations of leptin, insulin, non-esterified fatty acids (NEFA), adiponectin and resistin were assessed at baseline and the 3 months after RDN.

**Results:** There was a significant reduction in mean office systolic (168.75 ± 2.57 vs. 155.23 ± 3.17 mmHg, *p* < 0.001) and diastolic (90.68 ± 2.31 vs. 83.74 ± 2.36 mmHg, *p* < 0.001) BP 3 months after RDN. Body weight, plasma leptin and resistin levels and heart rate remained unchanged. Fasting insulin concentration significantly increased 3 months after the procedure (20.05 ± 1.46 vs. 29.70 ± 2.51 uU/ml, *p* = 0.002). There was a significant drop in circulating NEFA at follow up (1.01 ± 0.07 vs. 0.47 ± 0.04 mEq/l, *p* < 0.001). Adiponectin concentration was significantly higher after RDN (5,654 ± 800 vs. 6,644 ± 967 ng/ml, *p* = 0.024).

**Conclusions:** This is the first study to demonstrate that RDN is associated with potentially beneficial effects on aspects of the adipokine profile. Increased adiponectin and reduced NEFA production may contribute to BP reduction via an effect on metabolic pathways.

**Clinical Trial Registration Number:** NCT00483808, NCT00888433.

## Introduction

Resistant hypertension (RH) is a condition characterized by elevated blood pressure (BP) that persists despite the use of a diuretic and at least two other antihypertensive medications. Patients with RH are at an increased risk of a future cardiovascular events compared to those whose BP is controlled (Bangalore et al., 2014; Irvin et al., 2014). Whilst there are many classes of antihypertensive drugs available today, successful reduction in BP remains difficult. To combat the problem of treatment resistance to medications, renal sympathetic denervation (RDN) has been explored clinically as an alternative approach.

While epidemiological studies have clearly established that high BP is linked to cardiovascular disease, it is also known that the risk is further increased by the addition of obesity. Visceral obesity is highly prevalent among RH patients. The pathophysiology of obesity-associated hypertension is complex and characterized by activation of the sympathetic nervous system (Esler et al., 2006), renin-angiotensin system (Boustany et al., 2004), endothelial impairment (de Jongh et al., 2004) and other mechanisms.

The view that adipose tissue is merely a repository of stored lipids has been revised over recent years. As many as 50 different substances have been discovered to be synthesized by adipocytes, many of which play a role in more than lipid metabolism. In fact, adiponectin, resistin and leptin, mainly, but not exclusively, synthesized by adipocytes, have been implicated in BP control. For example, it has been shown in experimental models of obesity that increase in leptin, associated with body weight gain, is followed by an elevation in systolic and diastolic BP after 12 weeks of high fat diet (Simonds et al., 2014). The administration of leptin antibody to the same mice resulted in a significant reduction in BP (Simonds et al., 2014). We have also demonstrated in our earlier studies that leptin levels are associated with renal sympathetic nerve activity (Eikelis et al., 2003), which can lead to increase in BP. On the other hand, both clinical and experimental studies have demonstrated that adiponectin levels are inversely related to BP: adiponectin levels fall with increasing BP and vice versa (Imatoh et al., 2008; Baden et al., 2013).

While the effect of renal denervation (RDN) on the reduction of BP, which is, at least partially, achieved via the reduction of sympathetic stimulation to the kidney, has been examined in the last couple of years, not much is known in relation to the effect of RDN on adipokines. The aim of the current study was to determine the effect of renal ablation on adipokine profile in obese patients with RH. In particular whether the potential effect of RDN on the adipokine profile is associated with the fall in BP in patients with RH.

## Methods

### Participants

Fifty seven consecutive patients with RH participating in our RDN program formed the cohort for this study. The cohort comprised patients participating in the Symplicity I and II trials and their selection criteria was described in the previously published papers (Krum et al., 2009; Symplicity et al., 2010). Briefly, selected criteria required evidence of uncontrolled systolic BP ≥160 mmHg on at least three antihypertensive drugs including a diuretic if tolerated. Patients underwent a complete medical history and physical examination, and were advised not to change their medications during the study unless medically required. Their baseline characteristics are presented in Table 1. Briefly, patients with RH and systolic BP (SBP) ≥140 mmHg were eligible for inclusion. Patients were on a stable antihypertensive medication regime for at least 3 months prior to enrolment. Baseline office and follow-up BP measurements were taken with an automated Omron HEM-907 monitor, and the average of 3 measurements is reported. All patients were studied at baseline and 3-month follow-up in a supine position after a standardized light breakfast. They were also asked to refrain from consumption of alcoholic beverages for at least 48 h before a study visit. The study was approved by the Alfred Hospital Human Research Ethics Committee and each participant provided written informed consent.

**Table 1:** Baseline characteristics of the study cohort.

**Characteristic**	
Number	57
Age – years	60.8 ± 1.49
Male – n (%)	37 (64.9)
Body mass index – kg/m^2^	32.6 ± 0.73
Systolic blood pressure – mmHg	168.8 ± 2.57
Diastolic blood pressure – mmHg	90.7 ± 2.31
Heart rate – beats/min	68.3 ± 2.2
**Medical history**	
CAD	25%
Obstructive sleep apnea	11%
Stroke	7%
Type 2 diabetes	30%
Number of antihypertensive medications – n	4.5 ± 2.7
**Type of antihypertensive medications**	
ACE inhibitors	53%
Angiotensin receptor blockers	77%
Calcium channel blockers	39%
Beta blockers	51%
Alpha blockers	16%
Diuretics	84%
Statin	65%

### Renal denervation procedure

The RDN procedure was approved by the Therapeutic Goods and Drug Administration in Australia. Bilateral RDN procedure was performed as described previously (Krum et al., 2009). Briefly, a radiofrequency catheter (Simplicity; Medtronic Ardian Inc., Palo Alto, CA) introduced into each renal artery via the femoral artery was used to ablate renal sympathetic nerves, during which time anxiolytics and analgesics were administered intravenously to minimize the pain.

### Laboratory analyses

Fasted blood samples were collected into chilled EDTA containing tubes and spun at 3,500 rpm for 15 min at 4°C. Plasma was aliquoted into clean tubes and stored in an −80°C freezer until analysis. Plasma adiponectin and resistin levels were quantified by immunosorbent assays (R&D Systems, Minneapolis, MN, USA), leptin and insulin by radio-immuno assays (Merck Millipore, Missouri, USA) and non-esterified fatty acids (NEFA) using an enzymatic colorimetric method (Wako Pure Chemical Industries, Ltd., Osaka, Japan) according to the manufacturers' instructions. Sample absorbencies for adiponectin, resistin and NEFA were recorded using a Benchmark Plus Microplate spectrophotometer (Bio-Rad Laboratories, Hercules, CA). A gamma counter (PerkinElmer, Inc., MA, USA) was used to measure gamma radiation for insulin and leptin assays.

### Statistical analyses

Statistical analyses were performed using SigmaStat for Windows Version 3.5 (Jandel Scientific, San Rafael, CA). Paired *t*-test was applied to data to compare the effect of RDN. Univariate correlation between the baseline BP and reduction in BP and physiological and experimental variables were evaluated using Pearson's correlation coefficient. Data are expressed as mean ± SEM. Statistical significance was set at *p* < 0.05.

## Results

### Demographics

Details of the study participants are presented in Table 1. The patient group comprised 20 females (35%) and 37 males (65%). As a group, the patients were overweight/obese with an average body mass index (BMI) of 32.6 ± 0.73 kg/m^2^. There were no differences in baseline BMI, BP or HR between genders. As expected, females had higher leptin (20.3 ± 3.4 vs. 9.4 ± 1.4 ng/ml, *p* = 0.006) and adiponectin (9,105 ± 2,323 vs. 4,131 ± 321 ng/ml, *p* = 0.013) levels.

Sixty five percent of patients (*n* = 37) were on statin medication. No differences were observed in baseline systolic or diastolic BP and HR between those participants that were on statin medication vs. those that were not. However, the absolute reduction and percentage change in systolic BP at 3 months follow up was significantly greater in statin users (data not shown).

### Blood pressure response

**Figure 1:**
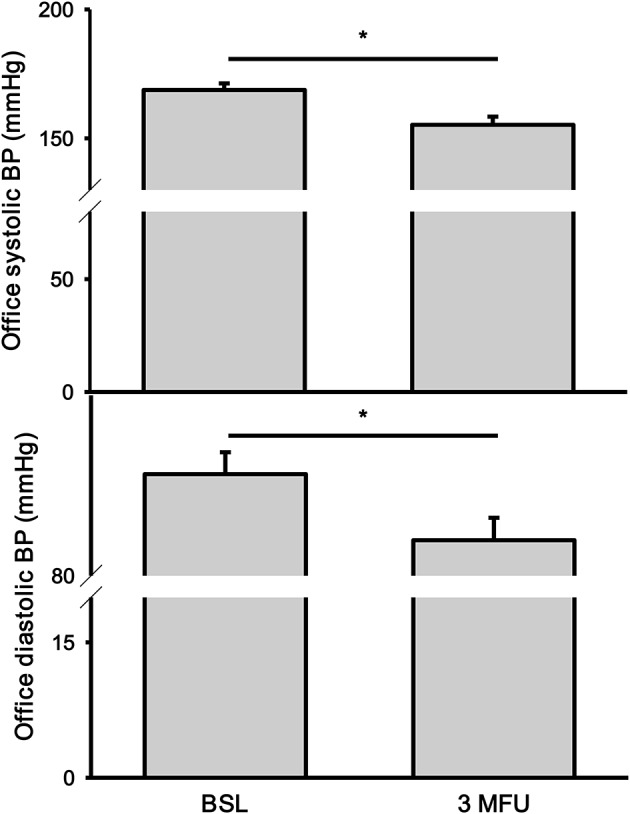
Office systolic **(top)** and diastolic **(bottom)** blood pressures (BP) at baseline (BSL) and 3 months follow up (3 MFU). Data expressed as mean ± SEM. ^*^p < 0.05.

The average reduction in SBP at 3 months follow up was 14 mmHg (168.8 ± 2.6 vs. 155.2 ± 3.2 mmHg, *p* < 0.001). Similarly, there was a significant drop in DBP (90.7 ± 2.3 vs. 83.7 ± 2.4, *p* < 0.001) (Figure 1). Heart rate remained unchanged.

### Adiponectin

As expected adiponectin had a strong inverse relationship with BMI at baseline (*r* = −0.398; *p* < 0.01). There was no significant correlation between adiponectin and BMI after the procedure (*r* = −0.292, *p* = 0.08). There was a significant increase in plasma adiponectin levels in patients after the procedure (5,654 ± 800 vs. 6,644 ± 967 ng/ml, *p* = 0.024) (**Figure 3**, top panel). There was no association between baseline adiponectin levels and the change in SBP at follow-up. Further analysis of the data revealed three patients that had high adiponectin levels (2 patients with high levels before and after denervation and one patient that had high adiponectin at follow-up). However, removing these data points from analysis did not change the significance of the finding (4,624 ± 546 vs. 5,437 ± 763 ng/ml, *p* = 0.025). No significant association were found between baseline adiponectin and BP reduction after renal ablation.

### Resistin

There was no change in plasma resistin levels in patients after RDN (7.06 ± 0.46 vs. 7.17 ± 0.36 ng/ml) (**Figure 3**, middle panel). Baseline resistin concentration did not have any association with baseline SBP nor with change in SBP after RDN.

### Leptin

**Figure 2:**
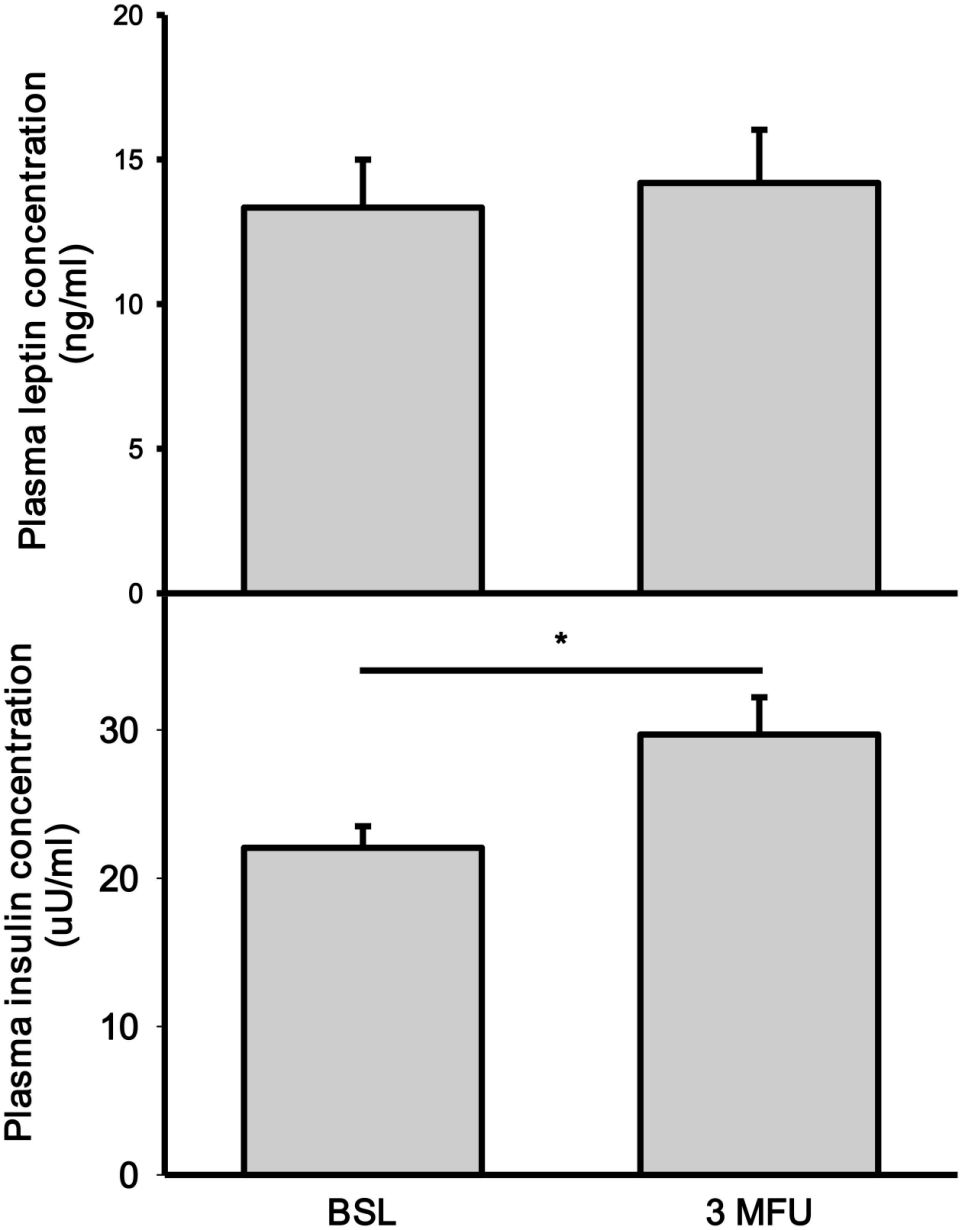
Plasma concentrations of leptin **(top)** and insulin **(bottom)** at baseline (BSL) and 3 months follow up (3 MFU). Data expressed as mean ± SEM. ^*^p < 0.05.

Leptin concentration was not significantly affected by RDN (13.34 ± 1.66 vs. 14.19 ± 1.84 ng/ml, *p* = 0.080) (Figure 2, top panel). There was no change in BMI in patients between the two visits (32.6 ± 0.81 kg/m^2^ at follow up). As expected, BMI was significantly associated with plasma leptin concentration (*p* < 0.001) both at baseline and follow up visits.

### Insulin

There was a significant increase in fasting insulin levels between baseline and follow-up visits (22.05 ± 1.46 vs. 29.70 ± 2.51 uU/ml, *p* = 002) (Figure 2, bottom panel).

### NEFA

**Figure 3:**
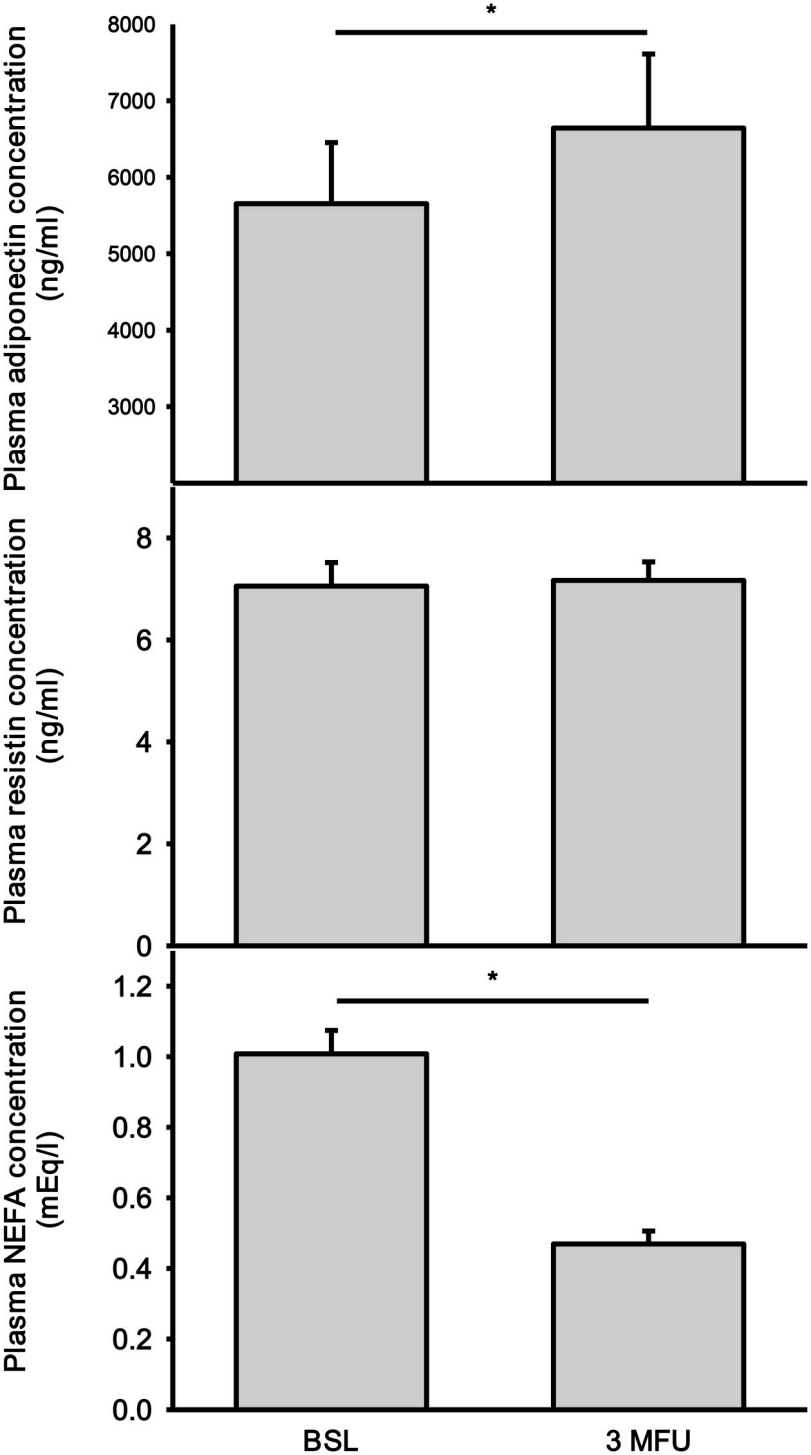
Plasma concentrations of adiponectin **(top)**, resistin **(middle)**, and non-esterified fatty acids (NEFA) **(bottom)** at baseline (BSL) and 3 months follow up (3 MFU). Data expressed as mean ± SEM. ^*^p < 0.05.

There was a significant reduction in NEFA concentration after RDN procedure (1.01 ± 0.07 vs. 0.47 ± 0.04 mEq/ml, *p* < 0.001) (Figure 3, bottom panel). This reduction was observed in all but 7 patients. No correlation was found between baseline NEFA levels and the magnitude of BP reduction.

## Discussion

This study was undertaken to determine whether RDN has any effect on the adipokine profile in mostly overweight/obese patients with RH. Here we report that RDN was associated with a significant increase in adiponectin levels at 3 months post-treatment. Adiponectin is almost solely synthesized by adipocytes. Its effects are known to be cardioprotective due largely to its anti-inflammatory and anti-atherogenic properties (Ohashi et al., 2012). Adiponectin is distinct from other adipokines as it is negatively correlated with body fat stores. Plasma adiponectin levels are reduced in people with obesity (Ryan et al., 2003). In the current study there were no changes in BMI between the two visits nor did we observe any significant changes in plasma leptin, which is a marker of fat stores. Therefore, we can conclude that the observed changes in adiponectin levels are not related to adipose stores.

Clinical studies suggest that adiponectin levels strongly correlate with BP, with hypoadiponectinaemia (low adiponectin levels) being a risk factor for hypertension. Baden and colleagues have demonstrated a significant negative correlation between adiponectin levels and BP in a group of healthy people over a wide adiponectin range (Baden et al., 2013). Adiponectin has been shown to exert its effects by stimulating the enzymatic activity of eNOS in endothelial cells. Such direct action of adiponectin on endothelial cells may represent the association of this adipokine and BP. Previous studies have demonstrated that adiponectin can decrease BP not only in hypertensive population but also in those with normal BP (Baden et al., 2013).

There is some evidence that sympathetic nervous activity can modulate adiponectin concentration. For example, Nowak *et al* demonstrated that 6 months treatment with the centrally acting sympatholytic agent, rilmenidine, in patients with essential hypertension was associated with increased adiponectin levels without changes in total body fat content (Nowak et al., 2005). On the other hand, cold exposure, which leads to heightened sympathetic activity, significantly reduced both adiponectin serum levels and adiponectin mRNA expression in subcutaneous, mesenteric and epididymal fat tissues in mice (Imai et al., 2006). It is well established that increased sympathetic drive is a hallmark of hypertension. Sympathetic activation, in its turn, has been shown to inhibit both synthesis and secretion of adiponectin. For example, administration of β-adrenergic agonists resulted in a reduction in adiponectin gene expression in human visceral adipose tissue (Delporte et al., 2002). We have demonstrated previously that RDN was associated with a significant reduction in renal noradrenaline spillover (indicative of renal sympathetic activity) (Krum et al., 2009) and muscle sympathetic nerve activity (Hering et al., 2013). Although sympathetic activity to adipose tissue was not examined in this study, we can only speculate that there was a reduction in sympathetic drive to adipose tissue after RDN which consequently resulted in higher circulating adiponectin.

On the other hand, there is also evidence that adiponectin might be involved in sympathetic regulation. Adiponectin was detected to be present in cerebrospinal fluid in humans which suggests that it may be able to act centrally to influence cardiovascular function (Kusminski et al., 2007). In support of this, both direct central and peripheral injections of adiponectin in anesthetized rats were associated with reduction of renal sympathetic nerve activity and BP (Tanida et al., 2007), therefore suggesting that BP lowering effects of adiponectin are at least partially mediated via suppression of sympathetic activity. While the nature of this study does not allow to state with certainty whether reduction in sympathetic activity after RDN caused adiponectin levels to increase or vice versa, it is probably the former, given that there was no change in weight at follow up visit. However, what might be the precise mechanism of adiponectin action in the central nervous system that would lead to changes in sympathetic outflow is not clear, but may be perhaps via leptin signaling.

In this study we also observed a significant increase in fasting insulin levels at 3 months after renal ablation. This finding was somewhat unexpected given Mahfoud et al. reported a significant reduction of fasting insulin levels in a similar group of resistant hypertensive patients at 3 months follow up accompanied by improvement in glucose metabolism (Mahfoud et al., 2011). Others have reported an improved glucose metabolism post-RDN procedure (Witkowski et al., 2011). It is possible that reported improvement is due to sympathetic inhibition which follows RDN (Hering et al., 2013). On the other hand, Miroslawska et al. reported no changes in either whole-body (peripheral) or hepatic insulin sensitivity in a group of resistant hypertensive patients at 6 month follow up after denervation despite a significant drop in BP (Miroslawska et al., 2016). It is important to note that in the current study we measured fasting insulin levels but did not evaluate insulin sensitivity and as such we cannot speculate whether there were changes in insulin or glucose status. It is possible; however, that renal ablation had a favorable effect on the recovery of the islet cells, which in turn resulted in an increase in insulin levels. Different medication regime and adherence, which was not assessed in the current study, might account for differences between data from different studies. The mechanisms by which RDN can affect insulin metabolism remain unknown and further studies should be designed to clarify the role of RDN on insulin sensitivity.

Resistin, while classified as an adipokine, is a protein produced primarily by macrophages in humans (Yang et al., 2003) and, when first discovered, was thought to provide a link between obesity and insulin resistance (Steppan et al., 2001). Later studies pointed to a role of resistin in the pathogenesis of hypertension (Zhang et al., 2010) possibly via inflammatory processes (Bokarewa et al., 2005). We did not see any changes in resistin concentration at 3 months after the procedure. Other studies have shown that hypertensive patients have higher resistin levels compared to normotensive individuals. We have not measured resistin levels in healthy individuals, however, compared to previously published reports, (Papadopoulos et al., 2005; Pantsulaia et al., 2007; Jamaluddin et al., 2012), resistin levels in the current cohort were not different from what is seen in healthy young people.

One of the explanations as to why resistin levels in our cohort were within the normal range could be due to the fact that resistin is affected by cholesterol lowering drugs such as statins, and that the 65% of patients in the current study were on statin therapy. For instance, treatment with atorvastatin for 6 months at a dose of 10 mg/day resulted in reduced resistin levels in patients with type 2 diabetes (Ichida et al., 2006). In the same study, the authors demonstrated that incubation of monocyte/macrophages with atorvastatin for 24/48 h led to a significant reduction in resistin expression as measured by qRT-PCR. Interestingly, no such effect of statin has been reported for adiponectin in hypertensive or type 2 diabetes patients (Kumada et al., 2003; Chu et al., 2008; Al-Azzam et al., 2013).

In this study we demonstrated a significant reduction in plasma NEFA levels post-RDN. NEFA is predominately derived from adipose tissue; and as such, NEFA concentration is increased in overweight/obese people (for review see Karpe et al., 2011). Circulating NEFAs are known to mediate adverse metabolic effects; with elevated NEFA at baseline serving a predictive value for type 2 diabetes. However, it should be mentioned that the increase in NEFA levels in obese individuals is not proportional to their BMI. In our study, the weight of the patients did not differ between the baseline and follow up visits. As mentioned above, leptin levels also remained unchanged. Therefore, the observation of reduced NEFA concentration post-denervation is intriguing. NEFA levels were reduced on average by half in 48 patients (out of 55 examined in this study).

Based on the results of clinical studies it would appear that there is a close association between NEFA and BP. For example, Steinberg et al. demonstrated that acute elevation in free fatty acids leads to a rise in systolic BP in lean insulin-sensitive patients (Steinberg et al., 1997). Furthermore, intravenous infusion of intralipid to healthy volunteers resulted in short term rise in BP and heart rate, suggesting altered central autonomic control (Stojiljkovic et al., 2001). NEFA can cross the blood-brain barrier where it can exert direct effects on sympathetic outflow to the muscle bed or other organs (Lam et al., 2005; Florian and Pawelczyk, 2009).

Reduction in NEFA could be due to reduction in the rate of lipolysis. One of the regulators of lipolysis is the sympathetic nervous system. In fact, about 40% of total free fatty acids released from adipocytes was shown to be attributed to sympathetic stimulation of adipose tissue (Hucking et al., 2003). Assessment of muscle sympathetic nerve activity and renal sympathetic nerve activity (using renal noradrenaline spillover technique) provided evidence of the reduction of the sympathetic drive to these organs after renal ablation (Krum et al., 2009; Hering et al., 2013, 2014). Therefore, it can be speculated that there may be a similar reduction of sympathetic activity to adipose tissue after RDN procedure. Further studies will need to address this issue directly.

It has been reported that adiponectin levels can affect NEFA concentration (Lavoie et al., 2009). Lavoie et al. demonstrated that there was an inverse association between circulating total and high molecular weight (HMW) adiponectin and the appearance of plasma NEFA during elevation of intravascular triacylglycerol lipolysis in healthy males (Lavoie et al., 2009). Due to the design of the study it cannot be concluded what was the nature of causal relationship between adiponectin and NEFA levels.

In conclusion, to our knowledge, this is the first study to demonstrate a favorable effect of RDN on adiponectin and NEFA levels, which may, at least in part, contribute to the reduction in BP. Whether adiponectin and NEFA changes subsequent to RDN are independent of the reduction of the sympathetic activation remain to be elucidated. While the clear-cut reductions in NEFA and increase in adiponectin after RDN in the absence of changes in weight, medication or other factors are suggestive of a potential causal association, in this observational study, we cannot establish the causation; nevertheless, the results are of interest and merit further investigation.

### Limitations

1. The current study did not include a control (sham) group.
2. While patients were instructed to keep their medications unchanged for the duration of the study, medication adherence was not confirmed.
3. It is possible that there was a Hawthorn-type effect and patients modified their behavior for the duration of the study. However, previous studies showed denervation resulted in cardiac remodeling (McLellan et al., 2015). Therefore, while we cannot be sure about the BP response, the results from these investigations do indicate clinical benefit in terms of change in cardiac structure which supports the observation that the BP reduction was of clinical significance.

## Author contributions

NE, conception of the manuscript/data acquisition/data analysis/drafting of the manuscript; DH, data acquisition/critical editing of the manuscript; PM, data acquisition/critical editing of the manuscript; JD, data acquisition/critical editing of the manuscript; LH, data acquisition/critical editing of the manuscript; AW, renal denervation/critical editing of the manuscript; EL, data analysis/critical editing of the manuscript; ME, critical editing of the manuscript; GL, data analysis/critical editing of the manuscript; MS, conception of the manuscript/data analysis/critical editing of the manuscript.

### Conflict of interest statement

The authors declare that the research was conducted in the absence of any commercial or financial relationships that could be construed as a potential conflict of interest. Drs. Walton, Krum, G Lambert, Esler, and Schlaich are investigators in studies sponsored by Medtronic. Dr. Walton is a proctor for Medtronic. The laboratories of Drs. Schlaich, Lambert and Esler currently receive research funding from Medtronic. Dr. Lambert has acted as a consultant for Medtronic and has received honoraria or travel support for presentations from Pfizer, Wyeth Pharmaceuticals, Servier and Medtronic. Professor Schlaich serves on scientific advisory boards for Abbott (formerly Solvay) Pharmaceuticals, BI, Novartis Pharmaceuticals, and Medtronic and has received honoraria and travel support from Abbott, BI, Servier, Novartis, and Medtronic.
